# Readmission and mortality in patients discharged with a diagnosis of medical observation and evaluation (Z03*-codes) from an acute admission unit in Denmark: a prospective cohort study

**DOI:** 10.1186/s12913-017-2156-9

**Published:** 2017-03-16

**Authors:** Kåre Melchior Hansen, Henrik Nielsen, Betina Vest-Hansen, Anders Møllekær, Reimar Wernich Thomsen, Ole Mølgaard, Hans Kirkegaard, Elisabeth Svensson

**Affiliations:** 1 Department of Clinical Epidemiology, Institute of Clinical Medicine, Aarhus University Hospital, Olof Palmes Allé 43-45, 8200 Aarhus, Denmark; 2 Research Center for Emergency Medicine, Aarhus University Hospital, Aarhus, Denmark; 3 Acute Medical Admission Unit, Aarhus University Hospital, Nørrebrogade, Aarhus, Denmark

**Keywords:** Z03, Emergency, Hospital admission, Readmission, Mortality, Epidemiology

## Abstract

**Background:**

We assessed the 30-day risk of readmission and mortality among patients receiving an International Classification of Diseases 10th edition diagnosis of medical observation and evaluation (Z03*) following admission to an acute medical admission unit (AMAU), stratified on any further specification of diagnosis during hospital stay.

**Methods:**

We used Central Denmark’s (Midt)-Electronic Patient Journal to identify patients with a Z03*-diagnosis among patients admitted to the AMAU, Aarhus University Hospital Nørrebrogade from April 2012 to March 2013, and noted any specification of diagnosis. Patients were followed from hospital discharge until death, emigration, or completion of 30 days follow-up.

**Results:**

Of 409 patients with an initial Z03* diagnosis at the AMAU, 55% (*n* = 226) received a more specific discharge diagnosis after transferral to other departments. Among patients discharged to home with a Z03*-diagnosis, 30% were readmitted within 30 days, while the corresponding figure was 23% for patients receiving a specific diagnosis (*p* = 0.06). In contrast, corresponding figures for 30-day mortality were 3% for Z03*-diagnosed patients and 10% for those who obtained a specific diagnosis (*p* = 0.003).

**Conclusions:**

Patients diagnosed with Z03* at hospital discharge have a substantially lower 30-day mortality, but a higher readmission-rate, compared to patients who obtain a specific diagnosis during the entire hospital stay.

## Background

Health care systems now face an ageing population with increasing multimorbidity in Denmark and elsewhere. It can be expected that an increasing proportion of acutely hospitalized medical patients will present with advanced age and comorbidities in the future [1].

Previous research shows that non-specific diagnoses, including ill defined (R*-coded) conditions and encounters for medical observation for suspected diseases and conditions (Z*-coded) are highly prevalent in patients in Denmark and other countries including Iceland and the UK, amounting to approximately 20% of all unplanned or emergency admissions [2–4]. Z*-diagnoses (85% of these being Z03*; “Medical observation and evaluation for suspected diseases and conditions” from chapter XXI in the International Classification of Diseases, Tenth Revision (ICD-10)) currently account for 17% of all patients admitted acutely to Danish medical departments [2, 5] and the use of Z*-diagnoses appears to be increasing [6]. Z03*-diagnoses include, quoting the World Health Organization (WHO) “persons who present some symptoms or evidence of an abnormal condition which requires study, but who, after examination and observation, show no need for further treatment or medical care” [7]. In a previous study covering acutely admitted patients in Denmark, Vest-Hansen *et al*. [5], observed that Z03*-coded patients were equally divided by gender, the majority was aged 60–79 years, and the majority did not have major comorbidities before the index admission. This indicates that these patients may be generally relatively healthy at baseline, compared to other hospitalized patients in this age group [5].

The large proportion of patients receiving unspecific diagnoses in Denmark and elsewhere underscores the need of further examination of this patient group and their clinical course and prognosis. Thus, the aim of our study was to examine 30-day readmission risk and 30-day post-discharge mortality among all patients discharged from an acute medical admission unit with a Z03*-diagnosis, and whether it differed if the patient received further specification of diagnosis during hospital stay or not.

## Methods

### Study setting and design

We conducted this study in one hospital in Aarhus, Denmark (population by April 1st 2012, *N* = 289.941 [8]). In Denmark’s tax-supported healthcare system, all citizens have equal access to specialist treatment and hospital care [9]. All Danish citizens receive a unique personal identification number (CPR number) at birth or immigration, used in all health databases and permitting unambiguous record linkage [10].

We created a cohort of all patients with a Z03 diagnosis, using Danish medical registries, which we followed with respect to mortality, re-hospitalization or end of follow-up.

### Study population

The study population included all Danish citizens aged over 18 years admitted to the AMAU, Aarhus University Hospital Nørrebrogade, Aarhus, Denmark, between April 1st 2012 and March 31st 2013 with a primary diagnosis of Z03* at discharge from AMAU, as registered in the Midt-Electronic Patient Journal (MidtEPJ). The MidtEPJ is a clinical care tool, and is related to an underlying patient administration system (PAS), which contains administrative data on every hospital contact, including time of admission, department, temporary location, and primary and secondary discharge diagnoses classified according to the International Classification of Diseases, Tenth Revision (ICD-10) since 1994 [7, 11]. Core data from the PAS are forwarded to the Danish National Patient Registry (DNPR). The MidtEPJ contains essential data not forwarded to DNPR, including temporary location codes, which in this study were crucial to identify patients at the AMAU.

Medical patients with suspicion of selected conditions (acute myocardial infarction (AMI), stroke, or pregnancy-related conditions) are referred directly to highly specialized departments, instead of being first admitted to AMAU.

### Classification of length of stay, departmental transfers, and specification of diagnoses

**Fig. 1:**
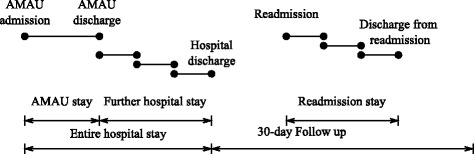
Length of stay

The length of an AMAU stay was classified as the time period in hours from AMAU admission to AMAU discharge, whether the discharge was directly to home or to another department (Fig. 1). The length of entire hospital stay was defined as the period from time of AMAU admission to the time of final hospital discharge, from AMAU or another department. We considered the patient discharged from hospital, if the time period between a department discharge and a new department admission exceeded 24 h.

Obtainment of a specific diagnosis during the entire hospital stay was defined as an initial Z03*-diagnosis achieved at AMAU discharge together with a non-Z03*-diagnoses achieved at final discharge from any department during further hospital stay.

### Readmission and mortality

We followed the patients from the date of hospital discharge (entire hospital stay) until death, emigration, or completion of 30 days of follow-up. Vital status was obtained through the Danish Civil Registration System (CRS), which includes exact date of death, updated daily [10].

We registered any readmission within 30 days after hospital discharge, using MidtEPJ.

### Comorbidity

Using information from PAS, we obtained information on the patient comorbidity burden at AMAU admission using the Charlson Comorbidity Index (CCI) [12]. We computed the CCI-score using all preceding primary diagnoses up to 5 years before the date of AMAU admission. We divided CCI-scores into low level (Index score 0), moderate level (Index score 1–2) and high level (Index score 3+). The CCI has been shown to have a high predictive ability in Danish medical registries [12].

### Statistical analysis

We described characteristics of the AMAU population. We estimated the median length of the stay at the AMAU and the entire hospital stay, and calculated the proportion of patients discharged to home, or with transferrals to another department. We calculated the proportion obtaining a specific diagnosis during the entire hospital stay.

We calculated the proportion of all patients readmitted within 30 days from hospital discharge, stratified by whether patients had obtained a specific diagnosis or maintained Z03*-diagnoses. We computed 30-day mortality overall and stratified by whether the patients obtained a specific diagnosis after transferral or maintained Z03*-diagnoses. We compared the proportions readmitted and 30-day mortality for patients obtaining a specific diagnosis or maintained the Z03*-diagnosis, using a one-sided z-test, with a significance level of 0.05.

Data were analyzed using SAS version 9.4

According to Danish legislation, individual informed consent or additional permission from the National Committee on Health Research Ethics is not required for studies based on information from registries and electronic databases with no patient contact taking place. In Denmark, a project, such as this being classified as an internal clinical quality enhancement project can gain access to data after being approved by the management of the clinical department, as this was.

## Results

Of the 4,916 patients admitted to AMAU, 8% (409) were discharged from AMAU with a Z03*-diagnosis. Median age of these patients was 69 years (interquartile range (IQR) 56–80 years), 51% were male, and 55% had no comorbidity (CCI-score = 0) (Table 1). The majority of patients (76%) were diagnosed with either “Observation for suspected disease or condition, unspecified” (Z03.9) or “Observation for other suspected diseases and conditions” (Z03.8).

**Table 1:** Baseline characteristics of the 409 patients discharged from the AMAU with a Z03*-diagnosis

	Number	Percent
*Gender*		
Female	200	49
Male	209	51
*Age (median years, IQR)*	69 (56–80)	
*CCI-score at index date*		
Low (0)	224	55
Moderate (1–2)	123	30
High (3+)	62	15
*Length of entire hospital stay in total (median hours, IQR)*		
	75 (17–187)	
*Length AMAU stay (median hours, IQR))*		
	14 (6–21)	
*Z03-subgroups at discharge from AMAU*		
Z03.0 - Observation for suspected tuberculosis	2	1
Z03.1- Observation for suspected malignant neoplasm	80	19
Z03.3 Observation for suspected nervous system disorder	5	1
Z03.4- Observation for suspected myocardial infarction	4	1
Z03.5- Observation for other suspected cardiovascular diseases	5	1
Z03.6 - Observation for suspected toxic effect from ingested substance	3	1
Z03.8 - Observation for other suspected diseases and conditions	36	9
Z03.9 - Observation for suspected disease or condition, unspecified	274	67

### Length of hospital stay, diagnoses, internal transfers

The median length of the AMAU stay was 14 h (IQR 6–21 h) and the median length of the entire hospital stay was 75 h (IQR 17–187 h).

**Fig. 2:**
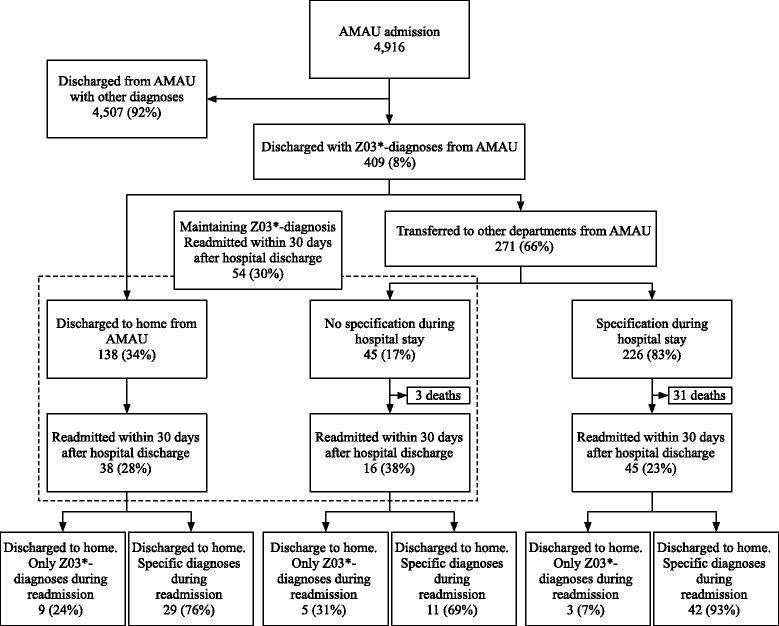
Proportion of all patients readmitted within 30 days from hospital discharge, stratified on maintaining diagnosis or further specification

Figure 2 shows the flow of patients from admission to discharge, and subsequent readmission and diagnoses. Of 409 patients with Z03*-diagnoses, 34% (138) were discharged directly to home from the AMAU. The remaining 271 patients were transferred, and 83% (226/271) obtained a specific diagnosis, corresponding to 55% (226/409) of all individuals with an initial Z03*-diagnosis at the AMAU.

The most common diseases among the 226 patients who eventually obtained a diagnosis were within ICD chapter J (respiratory diseases = 21%, most commonly pneumonia, COPD, or respiratory failure), chapter K (digestive diseases = 15%, most commonly alcoholic liver disease, cholelithiasis, or hepatic failure), chapter I (circulatory diseases = 12%, most commonly cerebral infarction, heart failure or atrial fibrillation and flutter), chapter C (malignant neoplasms = 11%, most commonly malignant neoplasm of bronchus and lung, or malignant neoplasm of pancreas) and chapter A (infectious and parasitic diseases = 10%, most commonly sepsis, gastroenteritis and colitis, or erysipelas).

### Readmission

Of the 138 patients discharged directly to home, 28% (38/138) were readmitted within 30 days after hospital discharge (Fig. 2). After readmission, 24% (9/38) were discharged with Z03*-diagnoses, while 76% (29/38) obtained a specific diagnosis. Of the 45 patients who maintained Z03*-diagnoses after transferral, 3 (6%) died during the hospitalization. Of the 42 patients eligible for readmission, 38% (16/42) were readmitted, and 69% (11/16) of these patients were discharged with a specific diagnosis. Thus, among all patients with a Z03*-diagnosis at hospital discharge, 30% (54/180) [95% CI 23.8;37.0] were readmitted within 30 days.

Among the 226 patients who obtained a specific diagnosis after transferral, 14% (31) died during further hospitalization. Of the 195 eligible for readmission, 23% (45/195) [95% CI 17.7;29.5] were readmitted, and here 93% (42/45) obtained a specific diagnosis. We observed a tendency towards a higher proportion being readmitted in the patients with Z03*-diagnosis at discharge compared to patients who obtained a specific diagnosis (*p* = 0.06).

### Mortality

**Fig. 3:**
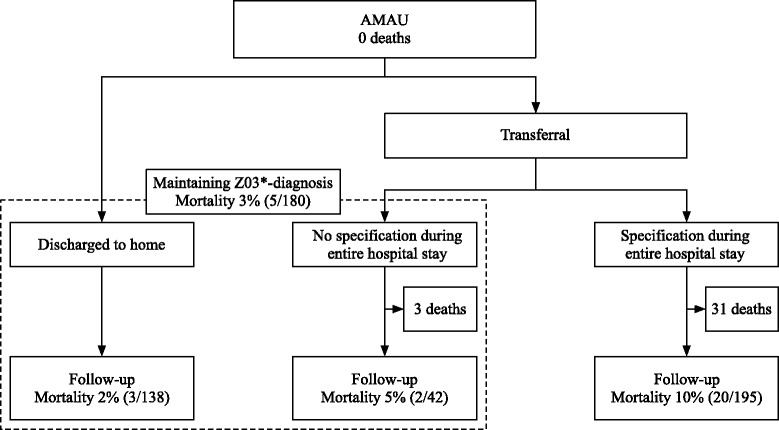
30-day mortality following hospital discharge after initial Z03*- diagnosis, stratified on maintaining diagnosis or further specification

The overall mortality during entire hospital stay and subsequent follow-up period was 14% (59/409) (Fig. 3). No patients died in the AMAU. Among all patients with a Z03*-diagnosis at hospital discharge, the 30-day mortality was 3% (5/180) [95% CI 1.2;6.3]. Among the patients who obtained a specification of diagnosis during the entire hospital stay, the 30-day post-discharge mortality was 10% (20/195) [95% CI 6.7;15.3]. The mortality was significantly higher in the group of patients who obtained a specific diagnosis after transferral compared to the patients who maintained Z03*-diagnoses (*P* = 0.003).

## Discussion

Patients diagnosed with Z03* at hospital discharge have a substantially lower 30-day mortality, but a higher readmission-rate, compared to patients who obtain a more specific diagnosis during the entire hospitalization stay.

Our findings corroborate and extend previous research on the use of unspecific Z03*- coding. The demographic characteristics of the patients in our study are in agreement with a national study by Vest-Hansen *et al*., while the median length of hospital stay was 3 days in our study compared to 1 day in their study [5]. Our study also yielded a considerable lower proportion discharged with Z03*-diagnoses (8%) compared to close to 20% based on previous Danish studies [2, 5]. Vest-Hansen *et al.* included all medical departments in Denmark including cardiac departments [5], and two out of three patients were actually being observed for myocardial infarction (Z03.4, “Observation for suspected myocardial infarction”). In our study patients suffering from symptoms of myocardial infarction would be referred to a specialized cardiac department before admission to AMAU. Therefore, this would be a plausible explanation for the inconsistencies in results.

In previous studies, the first admissions were primarily studied - building on this we followed the patients during the entire hospital stay, observing if the diagnosis changed. Overall more than half of the patients with Z03*-diagnoses at AMAU obtained a specific diagnosis before hospital discharge. The readmission rate was higher among the patients discharged with Z03*-diagnoses compared to the patients discharged after specification of diagnosis during hospital stay. The readmission rates were generally higher than in previous studies in acute medical admission or emergency admission [3, 13, 14]. Although not directly comparable to Z-coding, the R-codes of the ICD-10 also describe non-causative diagnoses, and it is suggested that patients getting R-codes is “in less need of acute care and (admissions) may be avoidable if appropriate services are in place” [3]. Could this also be true for the Z03*-diagnosed patients, not obtaining specific diagnoses later in their hospital stay? Further investigation in whether admittance is avoidable for the Z03*-patients is warranted, as this study is based on few patients.

The 30-day mortality rate among discharged patients who had maintained their Z03*-diagnosis was lower compared to the overall non-stratified mortality rate of 6% reported by Schmidt *et al.* after acute admission to a medical admission unit [2], while the patients with a specification of diagnosis was higher. The patients with the highest risk of death were those who after transferral obtained specific diagnoses. As early initiation of relevant treatment could reduce mortality, this group warrants further investigation.

Our study’s strengths include population-based design and complete follow-up for mortality. Use of routinely recorded diagnoses, collected independently of the study, reduced the risk of information bias. Data from MidtEPJ is transferred to DNPR, which is known as a valid data-source for covering acute admissions to medical departments [15, 16]. This study only includes patients with admission at AMAU and readmission at any hospital department in Aarhus. If a patient was transferred or readmitted to a department outside Aarhus, we would count them as discharged to home, but we assume this potential misclassification to be of minor influence. Potential coding inaccuracy is an important limitation.

The high and likely increasing proportion of imprecise Z-codes points to several potential healthcare problems. Firstly, it may suggest that some index conditions are improperly coded and underreported in the DNPR, which has direct impact on surveillance and research of important specific diseases. Second, frequent use of primary Z03*-diagnoses may hamper comparative hospital survival statistics such as hospital standardised mortality ratios [17] where survival is compared for frequent primary diagnoses that account for a large proportion of deaths. Third, it may point to the presence of a growing vulnerable patient group with frequent contacts to hospitals with unspecific symptoms. Fourth, it raises the probability that diagnostic efforts are insufficient, and people with undetermined diagnosis may have insufficient therapy and increased risk of readmission and death. Our study adds to our baseline understanding of the characteristics and flow of the many patients with initial imprecise Z-codes through further departments and admissions, and quantifies their risks of readmissions and mortality. Future clinically detailed studies should investigate the exact reasons for potentially avoidable readmissions and adverse clinical outcomes in these patients.

## Conclusion

Patients diagnosed with Z03* at hospital discharge have a substantially lower 30-day mortality, but a higher readmission-rate, compared to patients who obtain a more specific diagnosis during the entire hospitalization stay.

## Abbreviations

AMAU: Acute medical admission unit
AMI: Acute myocardial infarction
CCI: Charlson Comorbidity Index
CPR number: Personal identification number
CRS: Danish civil registration system
DNPR: Danish national patient registry
ICD-10: International classification of diseases, tenth revision
IQR: interquartile range
MidtEPJ: Midt-electronic patient journal
PAS: Patient administration system
WHO: World health organization
Z03*: Medical observation and evaluation
